# Address to the Graduating Class of the Atlanta Medical College

**Published:** 1890-04

**Authors:** Hooper Alexander

**Affiliations:** Atlanta, Ga.


VOL. VII. APRIL, 1890. No. 2.

### Original Communications.

### ADDRESS TO THE GRADUATING CLASS OF THE ATLANTA MEDICAL COLLEGE.

By HOOPER ALEXANDER, Esq., Atlanta, Ga.

*Gentlemen of the Faculty of Medicine:*

When I agreed to deliver this address I had not realized the difficulty attaching to the undertaking—a difficulty which would exist even to a speaker of unusual capacity, and which arises from the fact that at this time one is chosen to speak, in some degree at least, with the authority of an official instructor, while he himself is altogether without that pale into which these, his auditors, are candidates for admission. Any conscientious man, considering what ought to be said on an occasion like this, must of necessity perceive that he has assumed a duty entailing grave responsibility. Speaking therefore solely as I am moved by my recognition of that responsibility, I say to you in candor, that I am modest enough to have discovered my own unfitness for a task which I was, perhaps, rash to undertake. I mean not in any sense to depreciate the merit, or demerit, which may attach to the poor tricks of speech, or adorn the worthless baubles of rhetoric, but in honest confession of my own poverty of thought to magnify my office. Whether I can fill it even measurably well, or altogether ill, I invoke the charity of your superior intelligence, promising only to avoid any objects where ignorance might tempt me into platitude or heresy, and seeking always that my thoughts may come from the honest fountain of my own conviction, to find in coming the approbation of your learned and humane profession.

*Ladies and Gentlemen of the Audience:*

You who like me are of the uninitiate, and who are here only as witnesses before men to this conference of license by the sages of science on the tyros of medicine, to you, I address myself with something more of a sense of kinship. We have here, on the one hand, a body of men, whose long and illustrious devotion to our service has entitled them to our confidence, while their patient charity and steadfast courage have won our love; on the other, a number of aspirants, emulous to receive and wear the mantles which these must shortly lay aside. To-night, in your presence, by the sanction of your laws, those will deliver to these, under the solemn seal of this college, the charters of a franchise which will warrant them to demand the confidence of their fellowmen, when they profess to bestow the benefit of human science upon suffering humanity; the love and confidence which these have justly earned, demand our unqualified assent to the proposition logically following the delivery of these diplomas, that their recipients are fit to minister at the bedside of pain.

These men of science, being now about to bestow their apostolic succession, have chosen one from among you to add some words of authority on your behalf. You have heard me bespeak from the faculty, with that humility which is proper from us, their lenient consideration for the unlearned injunctions which you and I will lay on these, our brethren of late, how they demean themselves in their new offices. Let me now, in their hearing, invoke your sympathy as my fellowmen, when I assume, on your behalf and mine, to qualify our assent to these franchises by earnest adjuration as to our rights in the premises.

*Gentlemen of the Graduating Class:*

Let me make plain to you the ground from which I speak. I am not here to flatter or cajole. I despise the perfunctory office of a Commencement orator; words, words, words. There is a reason and a cause for this custom. The reason adhered to, the custom is dignified and excellent; the reason forgotten and the occasion made the cloak for the mere tricks of an orator, the custom ceases to be good and the speaker is but little more than a clown. The speaker who hides the occasion behind the drapery of his words does scant justice to the intelligence of those he is called upon to counsel, nor can he rise to the measure of his duty unless he is willing to address them frankly and honestly, courteously indeed, but firmly, man to man, as one citizen should address another touching their mutual rights and duties.

You have heard what I have said both to those in authority and to my fellow-witnesses of your promotion. Know ye that this occasion is not a hollow form nor these parchment sheets empty, meaningless and void. To these men, under whose teachings you have sat, the sovereign State has entrusted the franchise of instruction. Two million freemen, relying on the ability and integrity of these proven men have conferred on them the right to instruct and commission you. They exercise to-night a function of the law so high that no man may legally inquire of your fitness behind these testimonials. You have also heard me speak in deprecation of my task, the task of unlearned counsel to the learned. Believe me nevertheless that it is not inappropriate. On the contrary, it is altogether fit. For if I have thus far made my meaning clear, you may now perceive that three estates are gathered here participant in this ceremonial.

You are the first. The meaning of your presence here is this, that you now present yourself before the State, claimants for one of its highest franchises. Logically you assert by your claim that you are acquainted with the joints and members of this machine; that you have diligently studied and reasonably learned the secrets of its mystery; that you know at least the ordinary movements of its parts, their functions and the customary causes of disorder; that in general, you are competent to determine the seats of its distemper, remove the hostile invaders of its boundaries and restore it to its normal place. These judges here who have passed upon your claim you now call to witness of its truth. Beyond this you make certain promises, you agree to be honest and faithful in the duty you assume, that you will exercise in conscience, with charity and courage, the functions which the State permits and for which it lends you the assistance of its laws. Much more than this, and peculiarly important in the present time, you agree that as you have received the benefit of the past experience, you will not selfishly withhold, but will truthfully bring in and honestly surrender to the common stock whatever knowledge you acquire in the practice of your profession. These witnesses you invoke to testify hereafter how you keep your pledges.

This faculty is the second estate. Entrusted in the confidence of the sovereign with the right to adjudge your fitness and bestow the evidence thereof, they stand as the trusted deputies of the people. Authorized by their collegiate charter as the attorneys of the State, they bear testimony by these scrolls that you are worthy to be called. To them the State accords implicit trust, unqualified. They therefore are at once the fiduciary agents of the people, bound by every honorable tie to merit the confidence they receive, and the sponsors of your new baptism, well worthy to be vindicated by your faith and merit. You, stand, therefore, in double duty bound—first to the State which is the fountain of your honors, next to the faculty who now become the sureties of your work.

These others here, for whom and as one of whom I speak, are the third estate. We stand for the State itself, unskilled in science, bound of necessity to entrust the healthful regulation of our members to the skilled and learned, we have deputed these to make selection for us, and to-night are come in the name and behalf of all our fellow-citizens to hear the report of these judges aud witness their delivery to you of the charter of your calling. Finally and beyond this, I, as one of these, am permitted to add the more immediate counsel of the people to the mediate instruction they have hitherto given through the mouths of these trusted agents.

Hence my embarrassment. There are many things we would be glad to enjoin upon you. I select only two.

And first I say that high among the duties that you owe in virtue of your calling is the duty of courage. I do not believe that any man can be a good man who is not a brave man, least of all a physician.

Physical courage is requisite. Not the mere hardihood of a fool risking life with no end in view, but that cold calculating and deliberate courage which first weighs carefully the demands of duty, and having determined where that path lies, walks therein, unflinching, no matter what the peril. Are you the surgeon of an army? Have the courage to plant your table at the point most accessible to the battle and there discharge your offices though the bullets hurtle round your head like hail-storms. Does famine or pestilence threaten the people whom you serve? Send away your family if you please, but stand you firm. The physician who shrinks from that call is recreant to a trust and is worse than the soldier who abandons his colors. For you are soldiers. You hold the State's commission, and you have no right to any consideration touching your personal peril. It is a covenant, when you accept your office, that you will, if need be, interpose your bodies, a living bulwark, to stay the march of desolation.

All honor to the profession of medicine, for, to their eternal glory be it said, hitherto its members have had here no reproach. Whenever and wherever the yellow flag of pestilence has waved its horrid folds, your brethren have not shrunk from the contest to which it challenged.

It was a glorious and a beautiful custom, that when the gladiators entered the arena, in the presence of assembled Rome they made tender of their loyal devotion to the State by hailing its sovereignty incarnate in the Emperor with a "*Morituri te salutamus.*" And so ought you to enter the battle with death, as glorious hosts of your brethren have done before you; not indeed as the gladiators did, to stake your blood against the sesterces of crime; but calmly in the presence of an onlooking world, facing, if it be so, a certain death for the love of your fellow-men, turn your eyes, brave and patient, to our common sovereign God above, saying, "we who are about to die, salute thee."

And yet it is not alone in the exhibition of physical courage that your highest duty lies. There is a better courage which if you have not, you are not worthy. I suppose it must be called moral courage. Dare you show it?

Have you the courage, for instance, to stand for the rights ot men even in the person of those servants of crime (albeit more sinned against than sinning) whose bodies are forfeit to the State and whose labor it lets out for hire? Do you think that if official duty pointed you out as their peculiar guardian, you could defy wealth and power and set aside the demands of friendship without the hope of reward, (as you have good warrant to do in the example of one of your honored and honorable preceptors,) and day after day stand before the powers that be, and say, in firm persistence, "Lo, here are a thousand men and women, embruted it may be, the enemies of society, firebrands of crime, without moral sense or manly gratitude, a burden to the people, a menace to peace, outcast and sold into slavery, yet nevertheless wearing the form of men, made in the image of God, with human lives to be preserved and immortal souls to save, and they are not entreated even as the laws or the contract demands?" Can you do this and stand there and fight their fight for decency and humanity's sake, making no friends and many enemies? You may think the task an easy one, but I tell you it will try your courage.

Can you stand up firm in a municipality where all are striving hard for wealth, and in virtue of your calling as guardians of the public health, say to its government, "Here is a slaughter-house or yonder a factory, whose owners may earn scant livelihood or heap up conscienceless wealth, as the case may be, but beside them are thousands of the penniless and ignorant, living in squalid homes and inhaling the germs of disease?" Can you point out these nurseries of pestilence and clamor for the protection of the helpless, till even politicians dare not longer permit such evils?

Can you see your friend and neighbor, rich and powerful though he be, on the eve of persuading a town council to build a magnificent rainwater sewer for the reclamation of his waste lands, and rise up unasked and by the authority of your station as holders of these diplomas, say, "Gentlemen, you are doing wrong; I care not how much this work may enhance this citizen's wealth; you can put this money down in sewers in less favored though more populous sections, where it will carry away the disease-breeding filth and garbage that is sapping the lives of a thousand little children?"

Can you do these things and lift your voices and be heard for human woe and human despair, and cease not to be heard, persistent as long as wealth is tempted to "grind the faces of the poor?" You may think the tests are simple. If so, wait till your judgment is no longer formed in the enthusiasm of youth, but has borne the subtle contest with those "which devour widows' houses and for a pretence make long prayers."

And yet there is still another form in which your courage must be tested.

The architect who is not brave enough to say, "I cannot build the desired arch," is guilty of the blood that is crushed by the crumbling wall. The lawyer who dares not say "You can not safely evade this law," is answerable for the consequence, whether he has allowed his client to taint his deed with usury or sell a thing which the law forbids. The minister who will not say to the inquiring soul "Great is the mystery of godliness," but seeks rather to darken counsel with the precepts of sect, takes peril of his own salvation.

So you: I doubt not you join me in the illustrations: look well to your own sand-house. Be brave enough to say to your patient, "You need no medicine," or "You shall not humor this folly," or, if needs be, to say, "Sir, I cannot safely advise you; I recommend a wiser physician." Ah, gentlemen, there is the test supreme, the confession of ignorance. But, as a citizen, I tell you that you cannot be honest physicians or honorable men, unless you can acquire the courage frankly to own it when you do not understand the case. Nevertheless, I tell you if you conceal your ignorance and the patient die, you are worse than the assassin who purposely takes life for money.

But I said I would tell you of another duty than courage. Do you know what charity is? If not, can you learn?

I may not be able to tell you what charity is, but I can tell you some things that it is not. It is not the careless gift of money to the poor; it is not the giving of balls to the rich where the price of the tickets, less the cost of the favors in the german, finds its diminishing path into the mouths of the hungry; it is not the balancing up of a robbery by devoting a tithe of the proceeds to the robbed; it is never the humiliating conference of favors. Neither will you earn the recompense of the charitable by simply being able to boast that you are doing a large "charity practice." Do you remember the infinite scorn with which Christ taxed those who made the commandments of God of none effect by the frozen formalities of tradition; how he buried them beneath contempt for their selfish emasculation of the fifth commandment, "Moses bade you honor your father and your mother, but you say that whosoever shall say unto his father or his mother 'it is a gift' by whatsoever you may be profited by me, he shall be free?"

Let me tell you, gentlemen, that there is no charity which does not spring from the perennial fountain of a large and liberal love. If you discharge your duty in this regard, you will never hesitate to go where you are called, upon any consideration as touching the patient's ability to pay, nor ever set it down to your own credit that you have ministered largely to poverty.

Did you ever stop to consider that the physician and the priest are the veritable successors of Christ? He came teaching the gospel and healing the sick. These were the only injunctions with which he sent his apostles forth. And so it came about that for centuries the physician and the priest were one. The priest does not sell his offices for money. Shall it be that the physician has chosen the worser part?

I may be wrong in the belief, but I doubt if ever a physician became successful in his calling, or ever can, who enters it for the money that is in it. Those whose names are the lustrous ornaments of medicine are those who through toil and want, nay through hunger even, have fought the good fight, taking profit if it came, but fighting for the love of men.

It is true the laborer is worthy of his hire, but it has ever been the boast of your profession that you do not work for money, that you receive an honorarium and not a fee; that by so much are you better than a trade. It is no concern of mine how you are to live. That is your affair. But I do say that having chosen your calling, you ought to live up to the boast, which is the essence of your claim to be a profession. Go where you are called without thought of the reward and withhold not your bounty from the needy, for the very reason that makes them need your bounty. Nevertheless I tell you that by this course I do not believe that you will suffer. Plant your duty on this high plane and you will find plenty to call who will be both able and willing to bestow the honorarium, nor will you find that the honoraria are less than the ledger accounts.

Gentlemen, I congratulate you that you are now come to the beginning of your studies, and I bid you with all my heart God speed in your work. May you add to the store of science.

